# The impact of alkaline earth oxides on Bi_2_O_3_ and their catalytic activities in photodegradation of Bisphenol A

**DOI:** 10.3906/kim-2101-30

**Published:** 2021-06-30

**Authors:** Ümran ÜNLÜ, Sevgi KEMEÇ, Gülin Selda POZAN SOYLU

**Affiliations:** 1 Chemical Engineering Department Engineering Faculty, İstanbul University, Cerrahpaşa, İstanbul Turkey

**Keywords:** Photocatalysis, alkaline earth oxide, Bisphenol A, degradation, UV-B irradiation

## Abstract

The BPA into wastewater has posed a threat to environment and human health. Hence, we aimed to eliminate BPA in a short time and with a rapid degradation rate from food wastewater. Herein, the effects of different alkaline-earth oxide doped with Bi_2_O_3 _nanoparticles on the photocatalytic degradation of bisphenol A were investigated. SrO-Bi_2_O_3_, CaO-Bi_2_O_3_, and MgO-Bi_2_O_3 _binary oxides were prepared by wet-impregnation method. The structural and optical features of catalysts were clarified BET, XRD, DRS, FT-IR, PL, and SEM techniques. The photocatalytic activities of catalysts were compared for different light sources. Considering that the characterization analysis and experimental results, the highly improved photocatalytic activity was mainly attributed to the effective structure of the SrO-Bi_2_O_3 _binary oxide and the strong alkali properties in the nanocomposite. Obviously, 5wt% SrO-Bi_2_O_3 _photocatalyst showed more excellent degradation performance and highest degradation reaction rate (0.21 mg l^–^^1 ^min^–^^1^) within 30 min. It was observed that the photocatalytic activity improved by the additive of alkaline oxide on Bi_2_O_3_.

## 1. Introduction

Bisphenol-A or BPA which can be synthesized organic compound, has the formula (CH_3_)_2_C(C_6_H_4_OH)_2._ Research shows that BPA is one of the chemicals with the highest production capacity than other chemicals throughout the world [1,2]. As mentioned in the world health organization, BPA is a chemical utilized chiefly as a monomer in the production of polymers such as epoxy resins and polycarbonate plastic (PC). Additionally, it has uses in polysulfone, polyacrylate resins, polyester, and flame retardants. Polycarbonate (PC) is thoroughly used in food contact materials like baby bottles, tableware, food containers, drink bottles, processing materials, and water pipes. Epoxy resins are utilized as preservative linings for a diversity of canned foods and drinks and as a coating on lids of glass jars and bottles (WHO, INFOSAN 2009) World Health Organization and Food and Agriculture Organization of the United Nations. BISPHENOL A (BPA) - Current state of knowledge and future actions. International Food Safety Authorities Network 2009; Information Note No. 5/2009 - Bisphenol A. [Online] https://www.who.int/foodsafety/publications/fs_management/No_05_Bisphenol_A_Nov09_en.pdf. [accessed on _____]. [2]. According to the researchers BPA can mimic the actions of estrogen, binding to the same receptor in the body. So, degradation of BPA is important status [3]. Bisphenol-A is known to be one of the notable EDC (endocrine disrupting compounds) and harmful to human health, agriculture, and environment. BPA has stable structure so, degradation of Bisphenol A is difficult [4–6]. Compounds such as BPA at low concentrations have been found to be highly toxic, poorly biodegradable, and present carcinogenic properties.

Pollutants like BPA effecting the environment are serious problem in improving countries. Furthermore, the rising population and increasing requisitions for water resources cause that there is a constantly growing in this problem. BPA is a substance that affects human health and there is a problem such as global warming in our world, so the treatment of wastewater has become very important. Development of environmentally friendly methods for removal of BPA is one of the most relevant issues in the field of reaching the clean water. Therefore, various methods like chemical oxidation [6–8], physical elimination development [9], biodegradation [10], adsorption [11], and photodegradation have been developed for the degradation of BPA [12].

Previous studies have shown that photodegradation gives good results. So, heterogeneous photocatalytic oxidation has been seen as potential effective technique to deteriorate environmental pollutant. With this in mind, we prefer heterogeneous photocatalytic degradation which is more advantageous than the other methods since it is low cost, reusable, provides complete degradation, and it is eco-friendly method. 

BPA can be co-existing with other contaminants, so more selective catalysts should be used. During the study we used alkali metal oxides on bismuth oxide. The alkaline earth metals used in this study are often white in color, soft, and workable. In addition to being less reactive (prone to reactions) than alkali metals, their melting and boiling temperatures are also lower. Ionization energies are also higher than alkali metals. SrO impregnated with different proportions on Bi_2_O_3_ showed great selectivity and was successful in a short time. In addition to these different catalysts were used as the main catalyst [13–15]. When the previous studies were examined, titanium oxide was used many times as the main catalyst, as it has an extended band gap [14–17]. In particular, Bi_2_O_3_ has been used since it is a nontoxic and noncarcinogenic compound and has high photocatalytic activity with a bandwidth ranging from 2.0–2.8 eV [18–21]. 

The use of binary metal oxides as photocatalysts has been made widely for decades because of the fact that the morphological properties of the individual oxides can be changed due to the formation of new sites in the interface between the components, or by the incorporation of one oxide into the lattice of the other. They also found that this enhancement was attributed to gradually increasing shift of the conduction bands with increasing metal oxide contents, so resulting in a stronger reduction power of photogenerated electrons and promoting the improved photocatalytic activity.

In the current study, MxOy–Bi_2_O_3_ (M: Ca, Mg, Sr) photocatalysts with various loading of the metal oxides were prepared by impregnation method. The catalytic activities of synthesized materials were investigated by using UV irradiation. The complete degradation of pollutants such as BPA by photocatalytic methods is a promising solution for environmental problems and human health. In this study, the purpose is to explain the impact of parameters such as the use of different types of metal oxides and the percentage of metal oxides on the photooxidation of BPA. Another aim is to reduce the concentration of BPA. This is due to the fact that BPA has pollutants for the environment and harmful effects on human health. Moreover, the relationships between the catalyst morphologies and the photocatalytic activities were also investigated by using varied characterization methods such as scanning electron microscope (SEM), diffuse reflectance spectroscopy (DRS), Brunauer, fourier transform infrared (FTIR), Emmet and Teller (BET), and X-Ray diffraction (XRD).

## 2. Experimental and methods

### 2.1. Materials

In this experimental study, essential materials were supplied commercially and utilized without additional purification process. These materials are bismuth (III) nitrate pentahydrate (98%; Alfa Aesar Company, city, country), strontium carbonate (99%; Alfa Aesar Company), calcium nitrate tetrahydrate (Merck Company, city, country), strontium nitrate (Merck Company), magnesium nitrate hexahydrate (LACHEMA Company), ultra-pure water and bisphenol A (>= 99%, Sigma Aldrich). In addition to these, the others like nitric acid (65%), ethanol (absolute), acetonitrile (99.9%), and sodium hydroxide were bought from Merck Company. 

### 2.2. Catalyst synthesis methods

Co-precipitation method was utilized to synthesis Bi_2_O_3_ catalyst. 1.94 g of Bi(NO_3_)_2_.5H_2_O weighed by precision balance. In addition, 1.12 M 20 mL HNO_3_ (nitric acid-65%) solution and 0.2 M NaOH solution were prepared and kept in an ultrasonic bath for 15 min to ensure better dissolution. Finally, 20 mL of 1.12 molar HNO_3_ solution was added to 1.94 of bismuth, which was weighed and then kept in the ultrasonic bath for 15 min. The solution of resulting were mixed by magnetic mixer at the room temperature. Subsequently, 0.2 M NaOH solution by adding drop by drop into the bismuth (III) nitrate solution was provided to reach pH value 11 and then mixed for 2 h at 75 °C to make a homogenous yellowish mixture. Then this mixture was filtered as well as washed with distilled water and absolute ethanol several times. Obtained this matter was dried in a oven at 80 °C for 2 h, then calcined at 450 °C for 2 h.

In order that preparing of binary catalyst, impregnation method was used. This method is based on impregnation of metal oxide solutions onto pure bismuth oxide catalyst. When preparing solutions, attention was paid to the weight percentages of metal oxides present in the binary catalyst. Firstly, nitrous forms of metal oxides (Ca(NO_3_)_2_.4H_2_O, Mg(NO_3_)_2_.6H_2_O, Sr(NO_3_)_2_) and 0.2 M solution of 5% by weight in binary catalyst were prepared. These solutions were added dropwise to the powdered pure bismuth oxide catalyst to give a wet mixture and then dried at 105 °C. This process was continued until the solutions were finished. It was then dried in oven at 105 °C for 16 h and then was calcined at 500 °C during 3 h.

### 2.3. Catalyst characterization 

Total catalyst surface area of the catalysts was measured by nitrogen adsorption/desorption using a Quantachrome instrument. All catalysts were degassed under vacuum at 200 °C for 4 h.

Crystallographic structure of catalysts were determined by X-ray powder diffraction using CuKα radiation (λ = 1.54056 Å) with a Rigaku D/Max-2200 powder X-ray diffractometer. Before the analysis was run, the samples were gently granulated in an agate mortar to reduce the required orientation. Patterns were recorded at scan speed 2 degree of two-theta in the range of 10-90° 2θ. The average crystallite size (D_avg_) was computed using the Debye–Scherrer equation.

The powders were examined with a high resolution scanning electron microscope (SEM) (JEOL/JSM-a6335F) for possible differences in morphologies and size distributions of the powders. 

FT-IR spectra were examined by FT-IR spectroscopy (Perkin Elmer Precisely Spectrum One). KBr powder was used to prepare KBr pellets for samples. The samples were acquired as 100 scans with 4 cm **^–^** ^1 ^resolution

Optical energy gap of nano powders were carried out by a doublebeam UV-Shimadzu 3600 UV-vis-NIR spectrophotometer equipped a diffuse reflectance (DR) accessory.

The energy of band gap for the catalyst was evaluated by using the Kubelka–Munk formula with Tauc’s relation (Eq. (1)), which derived from DRS measurement.

(1)(hvF(Rα))1n=A(hv-Eg)(hvF(Rα))1n=A(hv-Eg)

In this expression, hν is the energy of a single proton, α is the optical absorption co-efficiency, E_g _is the optical band gap energy, A is constant for direct band gap transitions, the value of exponent parameter n denotes the nature of sample transition (n = ½ is used for the catalyst). R is the absolute reflectance value; F(R) is proportional to the absorption coefficient (α).

Moreover, photoluminescence (PL) spectra were obtained on a Cary Eclipse fluorescence spectrophotometer (Agilent Technologies, city, country). The catalyst was excited by a xenon lamp light source by 450 nm at the room temperature. 

### 2.4. Studies on photocatalytic activity

The experiment of BPA degradation was carried out over MxOy–Bi_2_O_3_ (M: Ca, Mg, Sr) binary oxide in a cylindrical quartz micro-photoreactor. Fifty mL of a 25 mg/L aqueous BPA solution was prepared and then 100 mg of catalyst was added in this mixture to initiate the reaction. Before exposure illumination, the solution was stirred in the dark for 1 h to establish an the adsorption-desorption equilibrium between the catalyst and the liquid. All reactions were conducted at ambient temperature under constant magnetic stirring and natural pH conditions. The photocatalytic activity of catalyst were compared using different light sources (UV-B, sunlight, and visible light irradiation). During the irradiation, sample was taken at regular intervals from the solution and filtered through a PTFE filter (pore size 0.45 mm for use total organic content (TOC) measurement (TOC-V, Shimadzu, city, country) and HPLC analysis. The analysis of BPA was performed by a HPLC (Thermo Scientific) using a C18 column. The mobile phase consists of a mixture of water and acetonitrile (40:60, v/v).

## 3. Results and discussion

### 3.1. Structural, morphological, and optical properties

The composition of the catalyst was determined using Thermo Elemental X Series ICP-MS. The actual weight percentages of the catalysts in the binary oxide catalysts were evaluated by ICP-MS analysis. The calculated wt% of the catalysts and the ICP-MS values were almost similar. Furthermore, ICP-MS values of the two representative catalysts clearly suggest that there may not be any noticeable differences between the calculated values and ICP values for the other weight percentages of metal oxides. 

**Figure 1:**
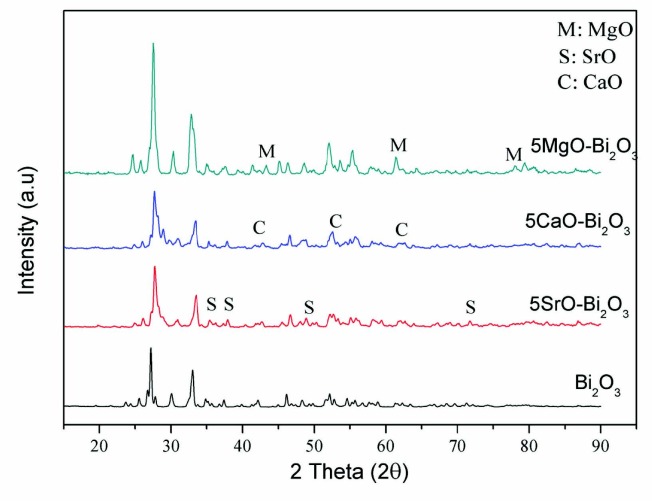
XRD patterns of Bi2O3, 5MgO-Bi2O3, 5CaO-Bi2O3, 5SrO-Bi2O3 catalysts.

The diffraction patterns of powder samples were examined to identify the phase structures. XRD patterns representing this are show in Figure 1. Monoclinic α-Bi_2_O_3_ nanorods from corresponding to JCPDS files (No. 41-1449) was observed as the main crystalline phase. In addition, tetragonal MgO (JCPDS 45-0946), cubic CaO (JCPDS 82-1691), and tetragonal SrO (JCPDS 48-1477) were detected in the XRD analysis. The crystallite sizes of the catalysts are presented in Table. 

**Table:** The crystallite size, specific surface area, band gap, morphology of materials, reaction rate constant, and Bisphenol-A (BPA) degradation efficiency over 30 min (%).

Catalyst	Crystallite size (nm)	SBET (m2g–1)	Band gap (eV)	BPA degradation efficiencies (%)		kr (mgL–1min–1)
Bi2O3	41	15	2.99	76	0.037	
5%MgO-Bi2O35%CaO-Bi2O35%SrO-Bi2O3	171915	71010	2.922.882.84	8488100	0.0360.0690.21	

After the impregnation of metal nitrate on Bi_2_O_3_, the lattice structure of Bi_2_O_3_ did not change. However, the average crystallite size of Bi_2_O_3 _changed with addition of metal oxide on Bi_2_O_3_. The crystallite size decreased with only the adding of metal oxide. After the XRD analysis, the crystallite sizes of samples were calculated as 41, 19, 17, and 15 nm for Bi_2_O_3_, 5MgO-Bi_2_O_3_, 5CaO-Bi_2_O_3_, 5SrO-Bi_2_O_3_, respectively. It has expressed that small crystallite size causes higher photocatalytic activity for the increased reactive sites and the promoted electron-hole separation efficiency [23].

Accordingly, the 5SrO-Bi_2_O_3 _binary oxideis expected to show higher photocatalytic activity due to its low crystallite size.

**Figure 2:**
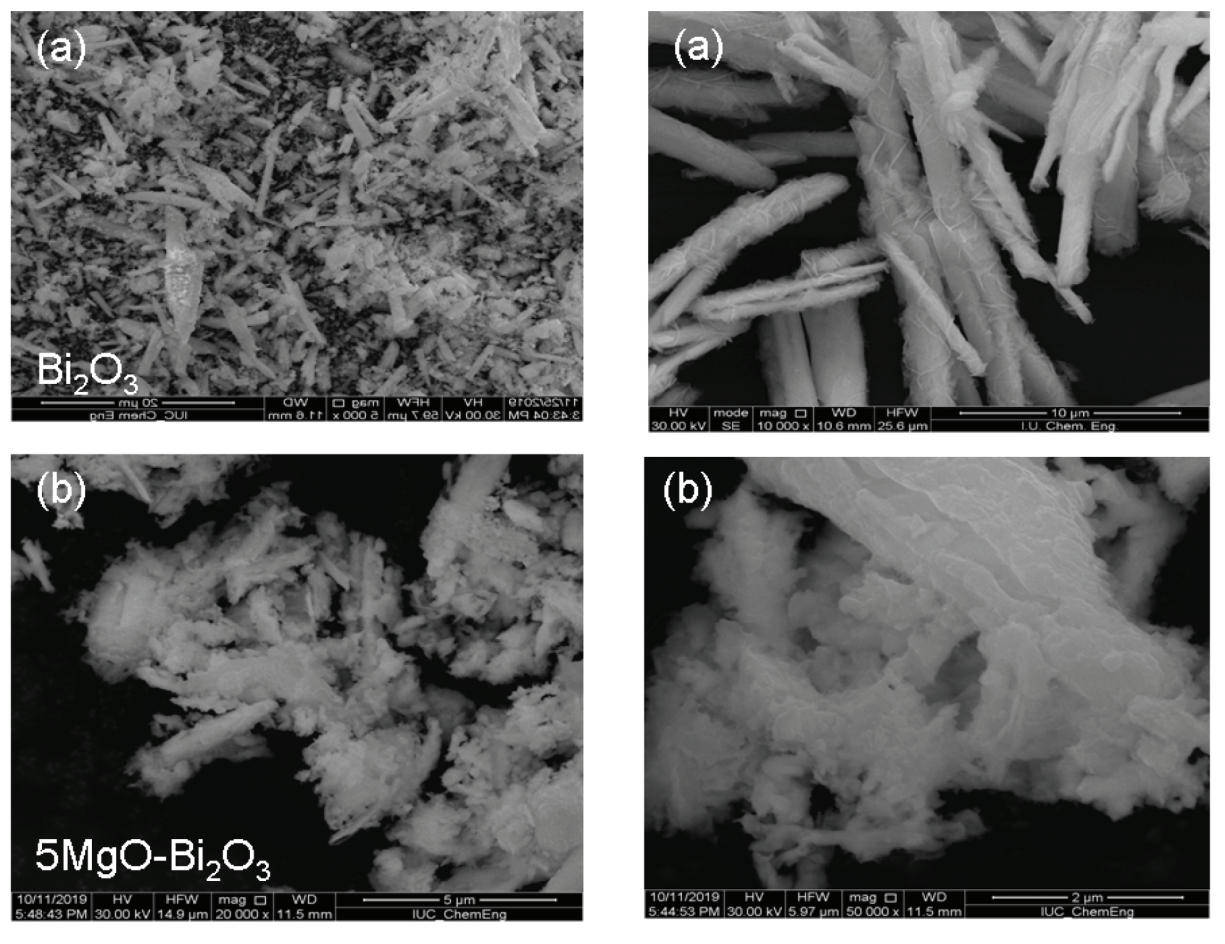
SEM images of (a) Bi2O3, (b) 5MgO-Bi2O3 catalysts.

**Figure 3:**
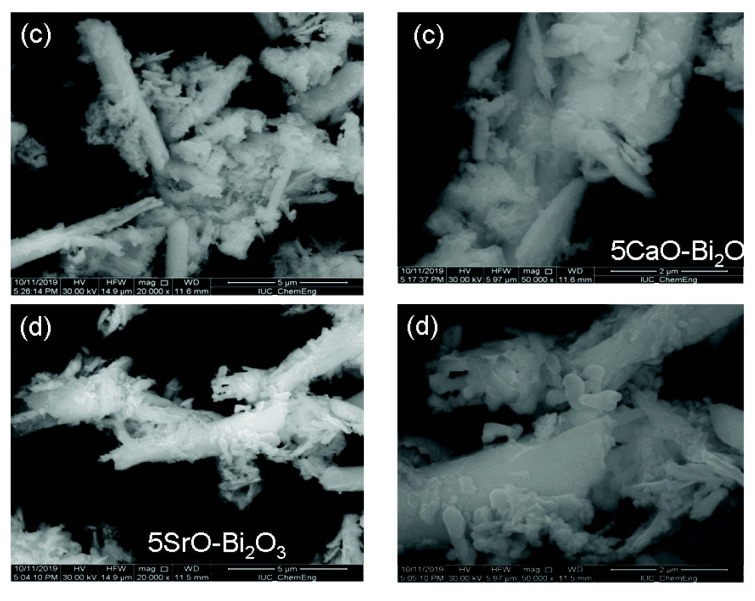
SEM images of (c) 5CaO-Bi2O3, (d) 5SrO-Bi2O3 catalysts.

The morphology and particle size of Bi_2_O_3_, 5MgO-Bi_2_O_3_, 5CaO-Bi_2_O_3_, and 5SrO-Bi_2_O_3_ were observed by SEM in Figure 2a, Figure 2b, Figure 3c, and Figure 3d, respectively. Figure 2a exhibits the SEM photograph of samples. The morphology of pure Bi_2_O_3_ is purely a nanorod. The metal oxide particles were observed on the surface of nano Bi_2_O_3_. 

5SrO/Bi_2_O_3_ catalyst has uniform morphology whereas; 5CaO/Bi_2_O_3_ and 5Mg/Bi_2_O_3_ are in irregular sizes and shapes. It was observed that SrO was distributed over the surface of nano Bi_2_O_3_ compared to CaO and MgO. The homogeneous distribution of the SrO structure on the nano Bi_2_O_3_ surface has played a role in improving the high photocatalytic activity.

**Figure 4:**
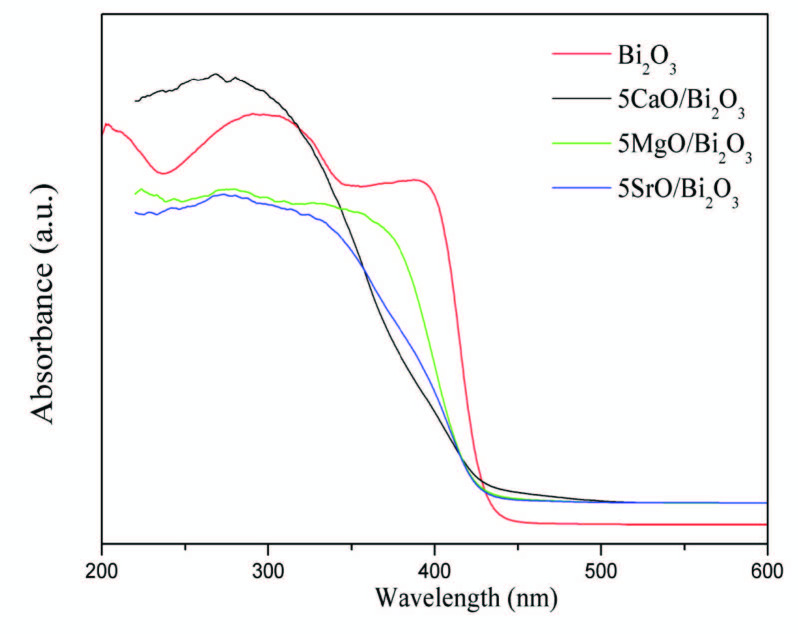
DRS spectra of Bi2O3, 5MgO-Bi2O3, 5CaO-Bi2O3, 5SrO-Bi2O3 catalysts.

**Figure 5:**
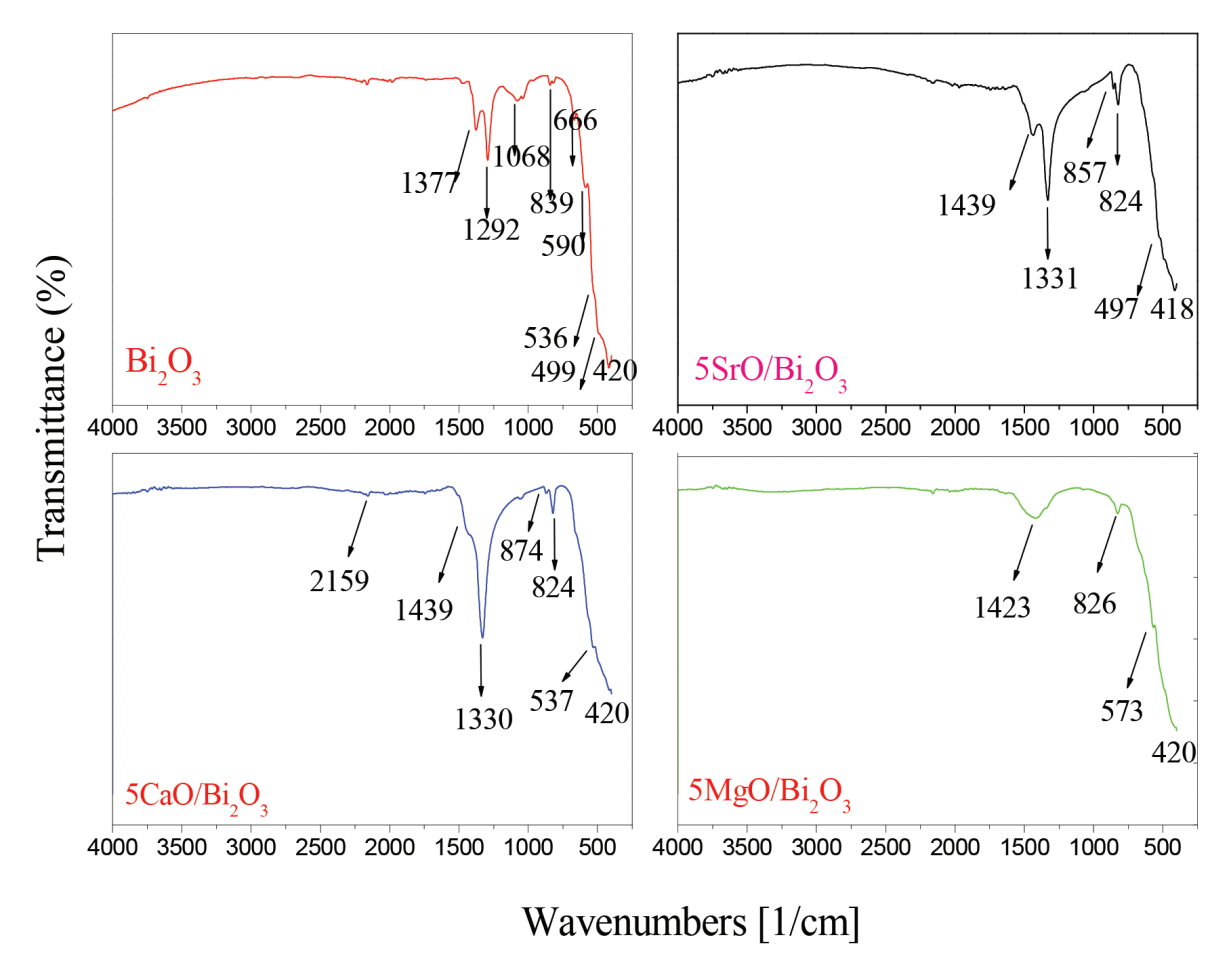
FTIR spectra of Bi2O3, 5MgO-Bi2O3, 5CaO-Bi2O3, 5SrO-Bi2O3 catalysts.

The change in the band gap energy of Bi_2_O_3_ with alkaline earth oxide loading was investigated using UV-vis diffuse reflectance spectroscopy. Figure 4 corresponds to UV-vis DRS spectra of the catalysts. The band gap energies of the samples were calculated from graphical extrapolation by using Tauc Plot ((hnα)^1/n ^= A(hn-E_g_)) adapted for Kubelka–Munk function [23]. The calculated band gap values are given in Table. According to the results, the band gap of pure Bi_2_O_3_ is 2.99 eV, whereas the band gap is decreased to 2.82 eV by the loading of 5 wt% SrO. The band gap of 5CaO/Bi_2_O_3_ and 5Mg/Bi_2_O_3_ are about 2.89, 2.92 eV, respectively. According these results, the loading of alkaline-metal oxide on Bi_2_O_3_ powder could significantly shift the optical band gap width E_g_. It can be caused that the optical properties of the samples were affected by the quantum size, which is a consequence of the extent of the electron delocalization. As seen SEM image, MgO structure covered to Bi_2_O_3_ external surface. Therefore, the diffuse reflectance spectrum of 5MgO/Bi_2_O_3 _is more different than others. Because of the band gap value of Bi_2_O_3_, we studied the photocatalytic degradation of Bisphenol-A under UV irradiation. Figure 5 shows the FTIR spectra from 4000–400 cm^–1^ for pure Bi_2_O_3_ and alkaline earth oxide additive Bi_2_O_3_ catalysts. Infrared spectroscopy technology is used to detect the presence of functional groups such as hydroxyl radical (•OH) adsorbed on the surface of synthesized nanoparticles. In the photocatalytic degradation experiments, the surface OH groups can not only embrace the photogenerated holes to form hydroxyl radical (•OH) but also serve as active sites for the adsorption of reactants [24]. The intensive signal at around 1430–1445 cm^–1^ for all alkaline-doped catalyst was attributed to the absorption of non-bridging O-H groups [25]. The concentration of the hydroxyl group was affected by the addition of alkaline oxide to Bi_2_O_3_.OH bending appears in the spectra for 5SrO/TiO_2_ and 5CaO/Bi_2_O_3_ catalyst at about 1439 cm^–1^. However, this peak was not observed after MgO loading. These results are in line with the activity results. 

In addition, the infrared spectrum of Bi_2_O_3_ was not observed any hydroxyl group. This result shows that the removing most of the adsorbed water from the surface of Bi_2_O_3_ with calcination process. In the spectrum, the band arises at 1300 cm^–1^ from the weak band of Bi–O–(NBO) bond in BO_3_ units for 5CaO/Bi_2_O_3_ and 5SrO/Bi_2_O_3_. This peak was not observed for 5MgO/ Bi_2_O_3_. The low absorption band at around 830 cm^–1^ seemed in FTIR spectra of samples is the stretching vibration of Bi─O bonds in BiO_6_ octahedral units [26]. The Bi─O bending vibration was observed at about 497, 537, and 830 cm^–1^ for Bi_2_O_3_. FTIR spectra of 5CaO/Bi_2_O_3_ revealed the existence of peak at 874 cm^–1^ which are the characteristic bands of Ca-O [27]. In addition, the peak of the Sr-O band was observed at 857 cm^–1^ in the spectrum [28].

As a result of the FT-IR study, it was understood that Sr^2+^ and Ca^2+^, except Mg^2+^, entered into the lattice of nano Bi_2_O_3_.

The ionic radiuses of Sr^2+^ (1.21 Å) and Ca^2+ ^(1.08)are larger than Bi^3+ ^(1.03 Å) but less than O^2^^– ^(1.31 Å). These ions can homogenously substituted or introduced into the nano Bi_2_O_3 _matrix to produce oxygen vacancies that accelerate the transition and nanocrystalline growth of Bi_2_O_3_ [29]. 

The occurrence of Bi-O-Sr prevents the transition of Bi_2_O_3_ phase and prevents the agglomeration of nano Bi_2_O_3 _particles. There is no evidence for an isomorphic settlement of Mg^2+ ^to the Bi_2_O_3_ structure observed due to the ionic radius of Mg^2+^(0.86 Å) are lower than Bi^3+ ^by adding Mg^2+^ to the nano Bi_2_O_3_ structure [30].

Photoluminescence spectroscopy is a widely used technique for characterization of optical and electronic properties of semiconductors and molecules. Photoluminescence can measure the purity and crystal quality of semiconductors and give some information oxygen vacancies, photo-induced charge carrier separation, and recombination processes surface states in nano-sized semiconductor materials. At low Pl density, the recombination rate of the electron hole is also low [31]. 

**Figure 6:**
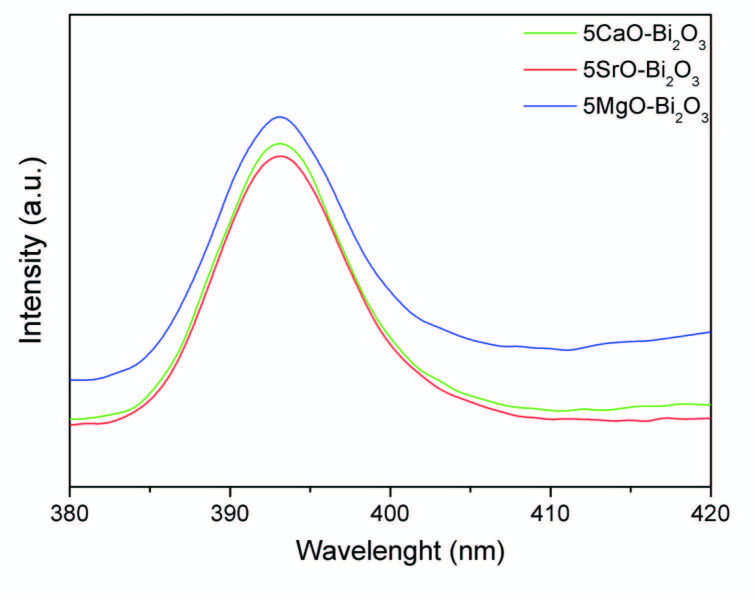
Photoluminescence spectrum of Bi2O3, 5MgO-Bi2O3, 5CaO-Bi2O3, 5SrO-Bi2O3 catalysts.

PL spectra of the catalysts are shown in Figure 6. It was understood that the PL emission spectra of the catalysts were at the same peak maximum but different densities. A strong emission peak at about 393 nm was obtained in the PL spectrum of the catalysts. It was determined that the density of the 5SrO-Bi_2_O_3_ catalyst in the emission spectrum was the lowest and this decrease showed the low recombination rate of the holes. As a result, it can be said that the 5SrO-Bi_2_O_3_ catalyst helps inhibit the recombination of electrons and holes and improve photocatalytic activity. 

### 3.2. Photocatalytic activity results

The photocatalytic activity of alkaline earth oxides loaded Bi_2_O_3_ was examined for Bisphenol A degradation. We based on Langmuir–Hinshelwood (L-H) kinetic model [32] in our experiments. It can be expressed by Equation 2 below: 

 ln(C_o_/C) = k_obs_t (2)

Assuming that C = C_o_ at t = 0 with low initial BPA concentration, where t is the given irradiation time and k_obs_ is the rate constant of the observed pseudo-first order reaction. After experiments, we calculated the photocatalytic degradation efficiencies and reaction rates for the different photocatalysts and the numerical values of gain are presented in Table.

The degradation efficiency (R%) of BPA was calculated by Equation (3):

(3)R%=C0-CC0

Firstly, we tested UV-B light and visible light as the different light source for degradation of BPA by using pure Bi_2_O_3_ and the degradation efficiencies of pure Bi_2_O_3_ were 76% and 47% for 30 min, respectively. The activity results show that although Bi_2_O_3_ can be activated under different irradiation, it is not sufficient for complete of BPA.

Moreover, we studied with SrO-Bi_2_O_3 _binary oxide prepared with varying SrO loadings. The degradation efficiency for the SrO-Bi_2_O_3_ binary oxide increased with the rise in the amount of SrOcharges to Bi_2_O_3_. It showed the highest percentage of BPA degradation (100%) in 30 min with a loading of 5% by weight SrO. However, there was no significant change in activity after this weight percentage and the value remained the same as the higher loading of SrO. The numerical values of gain showed that the structural and optical characterization of the samples and SrO dispersion on the surface affected with the loading of SrO in the binary oxide.

In addition, the performance of 5SrO-Bi_2_O_3_ catalyst was studied with UV-B light and visible light for BPA degradation. According the experimental results, binary metal oxide nanoparticles are more photoactivated under UV light than visible light irradiation. The degradation of BPA under visible light and UV-B light (64 W) showed 35% and 100% for 30 min, respectively.

In addition, the reusability of the 5SrO-Bi_2_O_3_ catalyst was studied on fresh dye samples (5 trials). 5SrO-Bi_2_O_3_, when used for the ﬁrst time, could degrade 98.52% BPA, with a small change (to 94.29%) in the efﬁciency when used for 5 times. This decrease in the efﬁciency for 5SrO-Bi_2_O_3_ catalyst resulted probably from the photocorrosion effect.

In this study, the photodegradation of BPA includes three steps. In the first stage, the transmission of electrons excited by photons emitted from the UV-B source from the valence band to the conduction band takes place. In the second stage, the holes formed due to the excitation process act as decomposing agent or combine with the surface hydroxyl species on the binary oxide to form the hydroxyl radical. In the last stage, the contaminant is attracted by the holes or hydroxyl radicals by the photons from the UV-B source.

**Figure 7:**
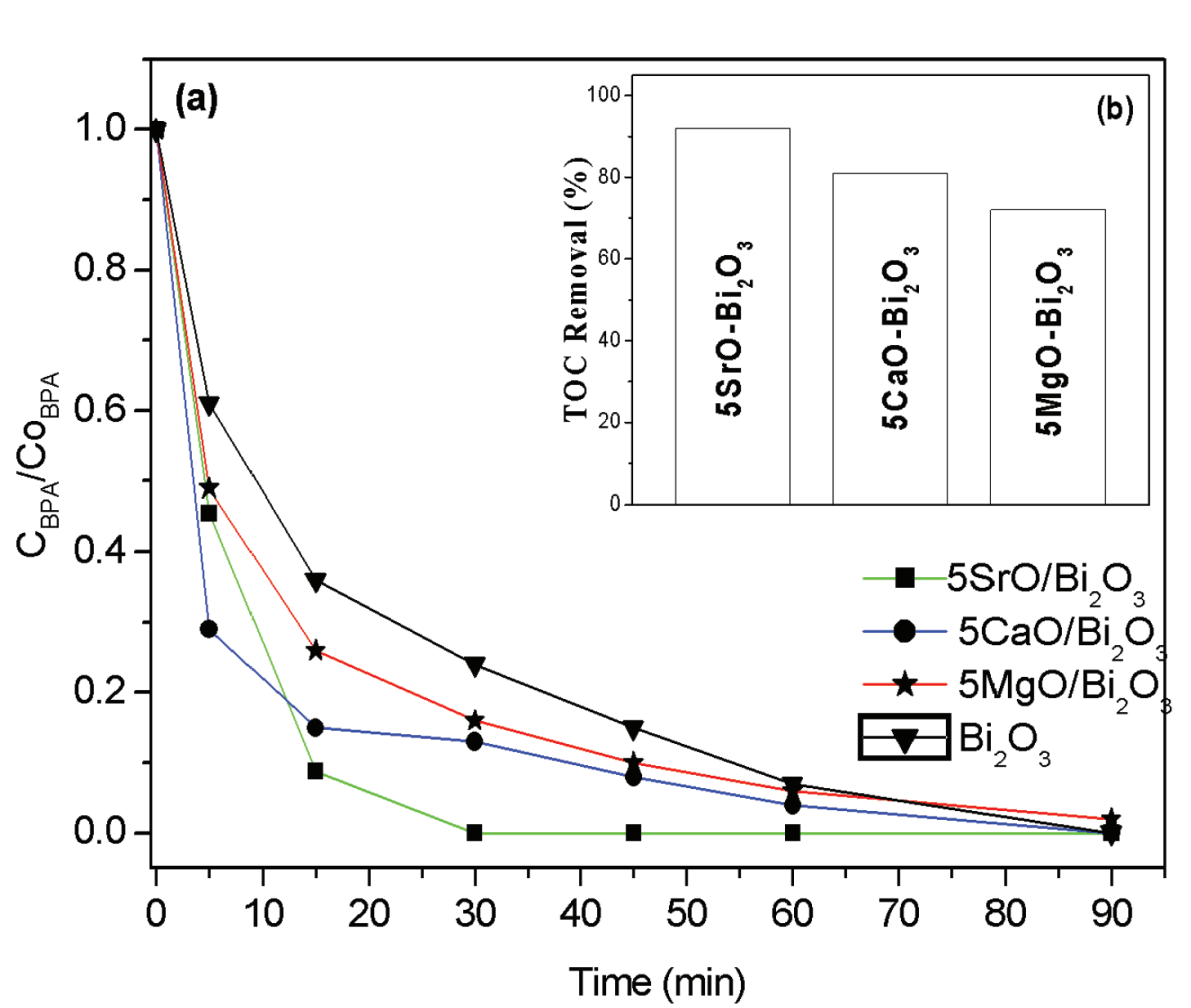
(a) Degradation activities of Bi2O3, 5MgO-Bi2O3, 5CaO-Bi2O3, 5SrO-Bi2O3 catalysts. (b) Inset shows the impact of surfactant on TOC removal in the BPA degradation.

Figure 7a shows the photocatalytic degradation of BPA in the pure Bi_2_O_3_ and alkaline earth oxide additive Bi_2_O_3_ catalysts under UV-B illumination at different irradiation times. When the degradation results are evaluated, the photocatalytic degradation rates of the BPA in 30 min with the binary metal oxides are sorted in descending order: 5SrO─Bi_2_O_3_ (ca. 100%), 5CaO─Bi_2_O_3 _(ca. 88%), 5MgO─Bi_2_O_3_ (ca. 84%), and Bi_2_O_3_ (ca. 76%). Obviously, 5SrO─Bi_2_O_3_ catalyst improved the degradation of BPA in a short time and with high efficiency compared to other catalysts and pure Bi_2_O_3_. The results show that •OH radical adsorbed on the catalyst was play an extreme role in the photodegradation of BPA. A small difference in the efficiency during the photocatalytic degradation of BPA can be elucidated by the free •OH radicals involved to a small extent in the photodegradation process [33].

The measured TOC removal results for the photodegradation of BPA with 5SrO─Bi_2_O_3_, 5CaO─Bi_2_O_3_,and 5MgO─Bi_2_O_3_ are shown in the Figure 7b. Twenty-five ppm BPA completely decomposed in 30 min with 5SrO─Bi_2_O_3_ binary oxide and 94% TOC removal was obtained in 30 min. It is clear that the total mineralization is completed in the presence of 5SrO─Bi_2_O_3_ catalyst.

The acid-base properties of the oxide catalyst can also affect the photocatalytic activity as well as its structural and optical properties.

As known, alkalinity of alkaline earth oxide increases from MgO to SrO. It seems that the activity changes in parallel with the alkalinity. As a result, the addition of strong basic alkaline oxide caused an increase in both the photocatalytic activity and the final conversion in oxidative degradation of BPA over catalysts. In addition to this result, the tendency of MgO to form larger particles on Bi_2_O_3_ surface also caused a decrease in BPA conversion. 

In the study, it was understood that the changes in surface area, band gap energy, and crystallite size were not as much as the changes in catalytic activity. These differences in activity are thought to be due to the active species on the surface of the oxide mixtures.

## 4. Conclusions

The effects of different alkaline-earth oxide doped with Bi_2_O_3 _nanoparticles on the photocatalytic degradation of bisphenol A were investigated. SrO-Bi_2_O_3_, CaO-Bi_2_O_3_, and MgO-Bi_2_O_3 _binary oxides were prepared by wet-impregnation method. The photocatalytic activities of the catalysts were compared for different light sources. Considering that the characterization analysis and improved photocatalytic activity results are mainly varied in relation to the effective structure of SrO-Bi_2_O_3_ binary oxide and the strong basic properties in the nanocomposite. Obviously, 5wt% SrO-Bi_2_O_3 _photocatalyst showed more excellent degradation performance and highest degradation reaction rate (0.21 mg l^–1 ^min^–1^) within 30 min. It was observed that the photocatalytic activity improved by the addition of alkaline-earth oxide on Bi_2_O_3_.

In this study, Sr^2+^ played an important role in reducing the crystallite size of nano Bi_2_O_3_. The small particle size of 5SrO-Bi_2_O_3_ and uniformly distribution of SrO on the pure nano Bi_2_O_3_ surface were very effective in the short time and complete degradation of BPA. The findings of this study elucidated an approach for the removal of BPA in the water through photocatalytic of degradation by alkaline-earth oxide doped with Bi_2_O_3_.
